# Spatial Structure and Activity of Sedimentary Microbial Communities Underlying a *Beggiatoa* spp. Mat in a Gulf of Mexico Hydrocarbon Seep

**DOI:** 10.1371/journal.pone.0008738

**Published:** 2010-01-15

**Authors:** Karen G. Lloyd, Daniel B. Albert, Jennifer F. Biddle, Jeffrey P. Chanton, Oscar Pizarro, Andreas Teske

**Affiliations:** 1 Department of Marine Sciences, University of North Carolina at Chapel Hill, Chapel Hill, North Carolina, United States of America; 2 Department of Oceanography, Florida State University, Tallahassee, Florida, United States of America; 3 Australian Centre for Field Robotics, The University of Sydney, Sydney, Australia; University of Wisconsin-Milwaukee, United States of America

## Abstract

**Background:**

Subsurface fluids from deep-sea hydrocarbon seeps undergo methane- and sulfur-cycling microbial transformations near the sediment surface. Hydrocarbon seep habitats are naturally patchy, with a mosaic of active seep sediments and non-seep sediments. Microbial community shifts and changing activity patterns on small spatial scales from seep to non-seep sediment remain to be examined in a comprehensive habitat study.

**Methodology/Principal Findings:**

We conducted a transect of biogeochemical measurements and gene expression related to methane- and sulfur-cycling at different sediment depths across a broad *Beggiatoa* spp. mat at Mississippi Canyon 118 (MC118) in the Gulf of Mexico. High process rates within the mat (∼400 cm and ∼10 cm from the mat's edge) contrasted with sharply diminished activity at ∼50 cm outside the mat, as shown by sulfate and methane concentration profiles, radiotracer rates of sulfate reduction and methane oxidation, and stable carbon isotopes. Likewise, 16S ribosomal rRNA, *dsrAB* (dissimilatory sulfite reductase) and *mcrA* (methyl coenzyme M reductase) mRNA transcripts of sulfate-reducing bacteria (Desulfobacteraceae and Desulfobulbaceae) and methane-cycling archaea (ANME-1 and ANME-2) were prevalent at the sediment surface under the mat and at its edge. Outside the mat at the surface, 16S rRNA sequences indicated mostly aerobes commonly found in seawater. The seep-related communities persisted at 12–20 cm depth inside and outside the mat. 16S rRNA transcripts and V6-tags reveal that bacterial and archaeal diversity underneath the mat are similar to each other, in contrast to oxic or microoxic habitats that have higher bacterial diversity.

**Conclusions/Significance:**

The visual patchiness of microbial mats reflects sharp discontinuities in microbial community structure and activity over sub-meter spatial scales; these discontinuities have to be taken into account in geochemical and microbiological inventories of seep environments. In contrast, 12–20 cm deep in the sediments microbial communities performing methane-cycling and sulfate reduction persist at lower metabolic rates regardless of mat cover, and may increase activity rapidly when subsurface flow changes.

## Introduction

In deep-sea hydrocarbon seeps, fluids that originate from thermal maturation of deeply buried fossil organic carbon seep into the upper sediment column, where they often solidify into methane-rich hydrates and may contribute to global climate forcing in episodic releases [1], [2]. Hydrocarbon seeps are not evenly distributed, but are found at localized hot spots dictated by the location of underlying conduits and fracture zones that vary through space and time [3], [4], [5], [6], [7]. Temporal shifts in hydrocarbon seeps result from relocation of subsurface conduits or from the temperature-driven destabilization of subsurface gas hydrates. Deep-source fluids and hydrates are transformed in surface sediments by highly active, benthic microbial ecosystems, which determine gas emissions and drive carbonate formation through methanogenesis, or sulfate reduction coupled to hydrocarbon oxidation [8], [9], [10]. The products of these anaerobic microbial processes, such as sulfide, incompletely oxidized organic compounds or dissolved inorganic carbon (DIC), are suitable substrates for sulfide-oxidizing *Beggiatoa* spp. These large, filamentous bacteria can be white, yellow, or orange and form extensive microbial mats with diameters of up to several meters, which cover the seafloor at methane seeps and hydrate sites in complex, patchy patterns [11], [12], [13].

**Figure 1:**
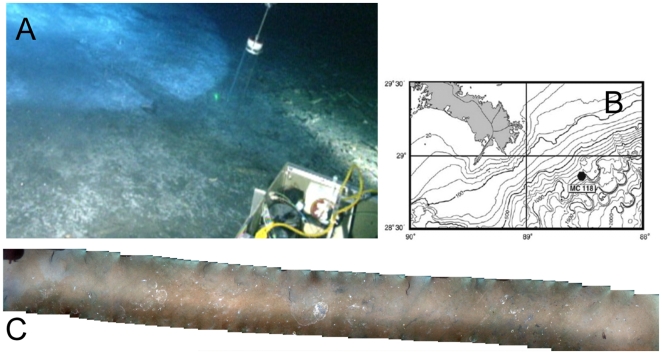
Locations and photos of sampling area. A) View of *Beggiatoa* spp. mat used for sampling. The mat covers the entire visible seafloor area of the photo; the white circle in the upper lefthand corner is the reflection of a light from the Johnson-Sea-Link submersible, B) Overview map of Mississippi Canyon block 118 (MC118) off the coast of Louisiana, and C) Mosaic image of seafloor a few tens of meters away from the large microbial mat in A; approximate width of the whole strip is 1.83 to 2.44 m given a submersible altitude of 3 to 4 m above the seafloor.

*Beggiatoa* spp. mats are often used as visual locators of active hydrocarbon seeps and seep-related microbial communities [6], [14], [15], but it is not clear how tightly coupled the presence of mat is to underlying seepage. Are the edges of a mat associated with diminished seepage rates that gradually transition to no seep influence in sediments some distance away from the mat? Or is the transition from seep-influenced to non-seep-influenced sediments and associated microbial communities abrupt, indicating a focused subsurface flow? Finally, since microbial mats cover only a fraction of the seafloor even at active seep sites, what can be inferred about the patchiness and distribution of seepage-associated microbial processes, such as methanogenesis, sulfate reduction, sulfate-dependent methane oxidation? We explored the relationship between geochemical activity measurements, and genetic analysis of the active microbial community with depth at different locations across a large (∼10 meter diameter, Fig. 1a) *Beggiatoa* spp. mat at a hydrocarbon seep in the Gulf of Mexico (Mississippi Canyon 118) (Fig. 1b). This habitat transect gives insights into the ecophysiology, activity, habitat preference, and diversity of these mostly uncultured microbial communities [16].

Establishing microbial activity with analysis of nucleic acids in the environment is difficult since DNA from inactive cells may be stable in cold anoxic sediments [17]. Therefore we used two forms of RNA obtained directly from bulk sediment to identify active microbial populations. In order to link sulfate reduction and methane oxidation/production as closely as possible to the corresponding gene expression pattern of the microbial community, messenger RNA (mRNA) of the genes for dissimilatory sulfite reductase (*dsrAB*) for sulfate reduction [18]and methyl coenzyme M reductase (*mcrA*) for methanogenesis and possibly also anaerobic methane oxidation [19], were reverse-transcribed and sequenced. Active bacteria and archaea have higher cellular rRNA concentrations relative to inactive bacteria and archaea [20], and rRNA content is positively correlated with independent measurements of cellular activity such as cellular protein levels [21] bromodeoxyuridine (BrdU) uptake [22], 3H-adenine incorporation [23], oxygen consumption [24], and chlorophyll a content and ^14^C-fixation rates [25]. Accordingly, RT-PCR-based studies have shown a significantly different population in marine sediments than were derived from PCR-based methods [22], [26], [27], [28]. For these reasons, RNA is more likely than DNA to reflect the active population, but preservation mechanisms may also exist for RNA in anoxic sediments or inactive cells, for instance low levels of 16S rRNA transcripts can persist in inactive methanogens at least a few hundred days [29]. A closer link to metabolic activity can be found in certain types of mRNA [30]. Transcription of *mcrA* is closely linked to metabolism in both *Methanococcus vanielii*, where *mcrA* has a maximum half-life of 15 minutes [31], and *Methanosarcina acetivorans*, where mutants can nonetheless arise that are capable of constitutive expression [32]. The expression of *dsrAB* genes is also coupled to sulfate-reducing activity in sediments [33] and in pure cultures of *Desulfobacterium autotrophicum* [34], although small amounts of constitutive expression during fermentation or thiosulfate reduction were also detected [34]. Since the small sizes of cDNA clone libraries often miss much of the microbial diversity present in the environment [35], we also checked selected samples using amplicon tag sequencing, where the V6 hypervariable regions of 16S rRNA genes undergo high throughput amplicon pyrosequencing to improve upon the sampling depth of clone libraries by at least two orders of magnitude and fully explore the microbial diversity [35].

Sequences of reverse-transcribed *dsrAB* and *mcrA* mRNA, as well as bacterial and archaeal 16S rRNA, were analyzed in conjunction with DNA-based V6-tag sequencing, porewater concentrations of methane and sulfate, radiotracer measurements of sulfate reduction and methane oxidation rates, and stable carbon isotopic values of methane to describe the spatial structure and activity patterns of sediment microorganisms with respect to *Beggiatoa* spp. mat location and hydrocarbon seep geochemistry at MC118.

## Results

### Geochemistry

**Figure 2:**
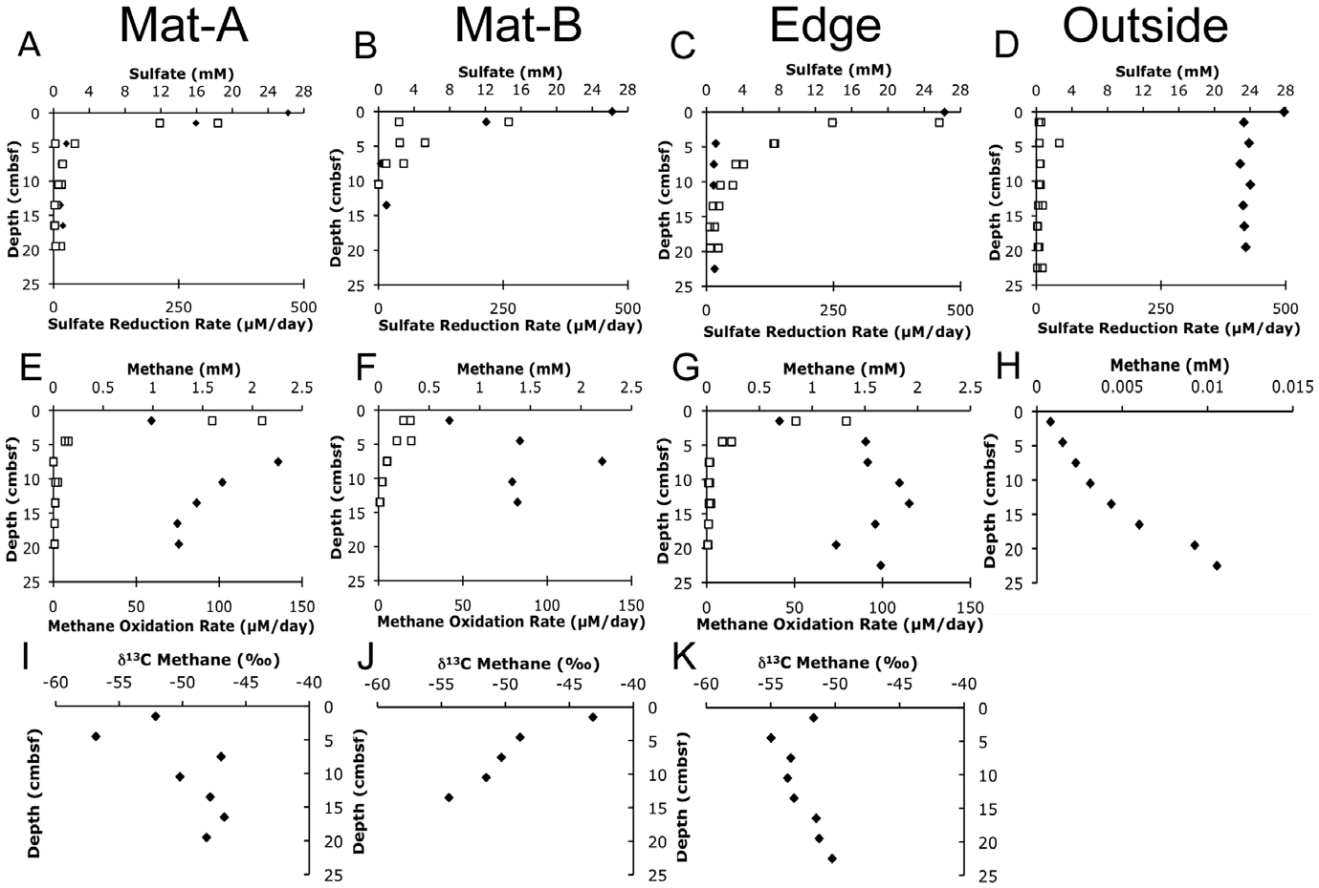
Geochemical measurements. Porewater measurements of A–D) sulfate concentrations (filled diamonds) and duplicate sulfate reduction rates (open squares), E–G) methane concentrations (filled diamonds) and duplicate methane oxidation rates (open squares), H) methane concentrations outside the mat with a smaller scale than the two other cores, and I–K) δ^13^C values for methane. Cores are from within the mat (A, B, E, F, I, and J), at its edge (C, G, and K), and outside the mat (D and H). Methane concentrations above ∼1.2 mM are lower limit estimations, since methane outgases to this value at normal atmospheric pressure. Only Mat-B, Edge, and Outside cores were used for microbiological analysis.

Steep sulfate and methane gradients were observed directly under the mat as well as at its edge (Fig. 2a–c). Sulfate was depleted to a relatively constant background concentration at 5 cmbsf and methane increased immediately below the seafloor. The decrease in radiotracer-measured sulfate reduction rates mirrored the sulfate concentrations. Although precautions were taken to minimize sulfide oxidation during sediment processing, the finite background concentration below 5 cm (0.7±0.3 mM) may be a sampling artifact; hence, measured sulfate reduction rates below 5 cm may overestimate in situ rates. The measured sulfate reduction rates cannot account for the shallow sulfate depletion depth: a 1-D, steady-state, reaction-transport model for sulfate using measured rates predicts that sulfate penetrates to >15 cm (Figure S1). This disparity could be due to upward advection of pore fluids at MC118, although we lack porewater chloride data to test this possibility. Lateral fluid flow is unlikely, given that sediments were compacted, but this is always a possibility [36]. Similar sulfate reduction rates were measured at the surface of another seep that has a similarly steep sulfate gradient [37]. Methane oxidation rates were much lower than sulfate reduction rates and also decrease with depth and sulfate concentration (Fig. 2e–g). Since methane concentrations were measured shipboard at 1 atm, any values above ∼1.2 mM (methane saturation at sea level) most likely underestimate methane concentrations at *in situ* pressure.

Methane has previously been shown to be both thermogenic and biogenic at MC118 [3]. The upcore ^13^C-depletion trends in the Mat-A and Edge cores also indicate methanogenesis (Fig. 2i–k) [38]. The ^13^C-enrichment of methane in the sediments of Mat-B suggests methane oxidation, since methane-oxidizing microorganisms have a kinetic preference for the lighter isotope as it diffuses upwards through the sediments [39]. Outgassing is unlikely to alter the methane δ^13^C profiles since this process has negligible isotopic fractionation [40]. Interpreting the relative locations of net methane oxidation or methanogenesis in the Mat-A and Edge cores is not possible, since ^13^C-enrichment was observed only in a single point at the surface for each core, and could reflect aerobic methane oxidation.

Just outside the mat, the sulfate concentrations did not decrease with depth (Fig. 2d). Likewise, the sulfate reduction rates were very low in this core. The methane concentrations were much lower than those of the mat cores, but the curved increase in methane with depth suggests oxidation of methane diffusing upwards from below (Fig. 2h) [41]. Outside the mat, methane concentrations were not high enough to accurately measure δ^13^C and methane oxidation rates were below the detection limit.

Sulfate and methane concentration fluxes across the first two depths were compared with total integrated rates of sulfate reduction and methane oxidation as a quality control check for measurements (Table S1). Most flux and rate measurements were in good agreement; only the second measurement of methane oxidation rates in Mat-B appeared to be largely underestimated.

### Bacterial 16S rRNA and *dsrAB* Transcripts

**Figure 3:**
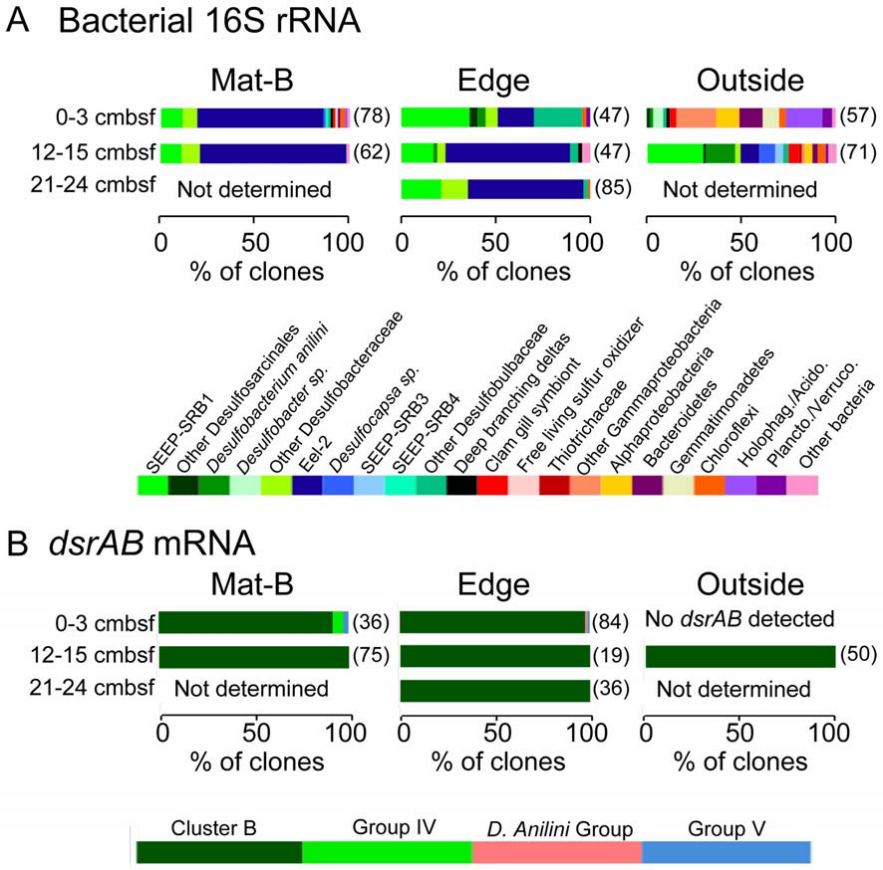
Bacterial community structure and stratification. Phylogenetic affiliations of (A) bacterial 16S rRNA transcripts, and (B) *dsrAB* mRNA transcripts at different depths across the mat transect. Shown are the percent of clones obtained from each group in the color coded bar graph legends. In A), shades of green and blue denote putative sulfate-reducing groups. Numbers in parentheses are the total number of clones analyzed, including full length and short reads. “Not determined” means that no amplification was tried.

The dominant sub-mat bacterial 16S rRNA transcripts were present at all sediment depths and position in the mat (center or near the edge of the mat) (Fig. 3a). Mat-A was not included in the molecular biological analysis. The majority of the 16S rRNA bacterial clones came from groups whose closest cultured relatives are SRB (Fig. S2). The clone libraries from sub-mat samples were dominated by phylotypes of the Eel-2 group, a sister group of the Desulfobulbaceae within the Deltaproteobacteria (Fig. 3a). The Eel-2 group has also been found at methane-rich areas off the coast of California [42], in the Black Sea [43], the Gulf of Mexico [15], [44], and a deep sea CO_2_ lake [45]. SEEP-SRB1 and other members of the Desulfobacteraceae also feature prominently, including the subgroup related to *Desulfobacterium anilini* which can degrade aromatic hydrocarbons. All cultured members of the Desulfobacteraceae oxidize organic carbon compounds completely to CO_2_. Members of the Desulfobulbaceae are also present; cultured members of this Family oxidize a wide range of carbon molecules incompletely. In particular, cultured members of the genus *Desulfocapsa* are able to disproportionate elemental sulfur. In the surface sediments outside the mat, bacterial 16S rRNA transcript composition changes abruptly to a diverse assemblage of phylotypes related to aerobic, microaerophilic or nitrate-reducing bacteria (Fig. 3a). Some of these aerobic groups that dominate the transcript libraries at the surface outside the mat, such as Alphaproteobacteria, sulfur-oxidizing Gammaproteobacteria, Acidobacteria, and the Bacteriodetes phylum, are also present at the surface within the mat, but in much smaller clone proportions relative to the SRB (Fig. 3a, Fig. S3). Likewise, SRB 16S rRNA transcripts are also present in surficial sediment outside the mat, but in much lower abundance relative to the aerobic groups. The bacterial community of the deeper sample outside the mat resembles the sub-mat community, and is dominated by SEEP-SRB1 and other members of the Desulfobacteraceae. Many of the aerobic and microaerophilic groups also persist in the deep sample as well, but in lower clone abundance relative to SRB (Fig. 3a, Fig. S3). The only group common to all samples was the Chloroflexi, which are commonly found in deep and shallow subsurface libraries [46]. Although the 0–3 cmbsf sediment samples were taken just below the bacterial mat, no sequences for *Beggiatoa* spp. were found, consistent with the frequently observed difficulty to amplify full-length *Beggiatoa* spp. 16S rRNA sequences from mixed environmental samples [47].

Similarly to the RT-PCR clone libraries, the V6-tag sequences (available from 12–15 cmbsf inside and outside the mat) were dominated by members of the Deltaproteobacteria (Fig. S4a). Deep samples within and outside the mat were similar to each other, and also had contributions from some groups that did not appear in RT-PCR clone libraries: Japan Sea Group 1 (JS1), Epsilonproteobacteria, Spirochaetes, Deferribacteres, Lentisphaera, OP8, and Actinobacteria, and a few others. V6-tag sequences are too short to allow further reliable phylogenetic identification [48].

Underneath the mat and at its edge *dsrAB* transcripts were recovered from all depths and the majority of them were related to the uncultivated Cluster B group [49] that is basal to the Desulfobacteraceae (Fig. 3b; Fig. S5). Other *dsrAB* transcripts are found only in the surface under the mat and include uncultured Group IV [50] and Group V [51], and *Desulfobacterium anilini*. No *dsrAB* transcripts of the Desulfobulbaceae were detected, even though they were present in the 16S rRNA transcript libraries. Primer bias most likely explains this result; one of the internal *dsrAB* primers used in this study had between three and four mismatches to cultured members of the Desulfobulbaceae (Table S2).

No *dsrAB* transcripts were detected from surface sediments outside the mat, despite nested amplification with multiple primer sets. Deeper sediments outside the mat yielded *dsrAB* sequences that were similar to those found under the mat, grouping with Cluster B (Fig. 3b).

### Archaeal 16S rRNA and *mcrA* Transcripts

**Figure 4:**
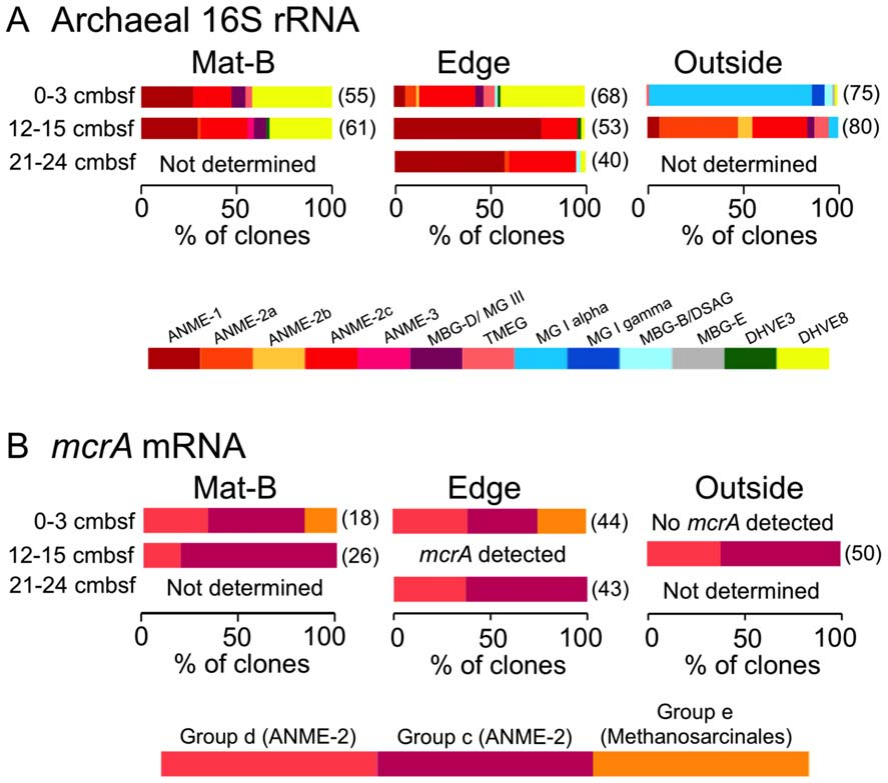
Archaeal community structure and stratification. Phylogenetic affiliations of (A) archaeal 16S rRNA and (B) *mcrA* mRNA transcripts at different depths across the mat transect. In A), shades of red and orange denote putative methane-oxidizing or methane-producing groups. Numbers in parentheses are the total number of clones analyzed including full length and short reads. “Not determined” means that no amplification was tried; “*mcrA* detected” means that *mcrA* was amplified but not sequenced.

At all depths underneath the mat and at the mat's edge, the majority of 16S rRNA sequences fall within the ANME-1b and ANME-2a and 2c groups, which are commonly thought to mediate sulfate-dependent anaerobic methane oxidation and are also found in net methane-producing sediments [52], [53], [54] (Fig. 4a and Fig. S6). The second most abundant sequence type, in all except the deep samples from the mat edge, are in the Deep Sea Hydrothermal Vent Euryarchaeota Group 8 (DHVE8) [55] within the DHVE II [56]. These sediment layers also contain 16S rRNA sequences from the Thermoplasmatales, Marine Benthic Group D (also called Marine Group III), and Marine Benthic Group B (also called the Deep Sea Archaeal Group). These uncultured archaea are commonly found in shallow and deep marine sediments [57].

Just outside the mat, the composition of the active archaeal community shifts to nearly exclusively Marine Group I at the sediment surface, and ANME-2a at 12–15 cmbsf (Fig. 4a). Marine Group I Crenarchaeota are the most abundant prokaryotic plankton in deep ocean water [58]. Genomics and physiology of a few species within Marine Group I have been studied using the naturally enriched candidate species *Cenarchaeum symbiosum*, an ammonia-oxidizing sponge symbiont, and the pure culture strain *Nitrosopumilus marinus*, an aquarium isolate capable of aerobic ammonium oxidation to nitrate chemolithoautotrophically [59]. A distantly related thermophilic representative has been cultured from a Yellowstone hot spring [60]. However, Marine Group I is a phylogenetically diverse group whose range of functions in the environment have not yet been fully explored.

For the archaea, taxonomic associations of the most commonly retrieved groups for the V6-tags generally supported the findings of the clone libraries. Deep samples within the mat and outside it were mostly composed of ANME-1 and ANME-2, with contributions from common benthic groups MBG-D and other members of the Thermoplasmatales (Fig. S4b). As with the bacterial V6-tag dataset, some common benthic groups were represented by the archaeal V6-tags that did not appear in the RT-PCR clone libraries: MBG-B/DSAG, Miscellaneous Crenarcheotal Group, and a few others in lower abundance (Fig. S4b). The DHVE8 group appeared in RT-PCR clone libraries, but not in the V6-tags, although the V6-tag sequences may not have been phylogenetically informative enough to distinguish this group.

Messenger RNA for *mcrA* was found in all samples except for the top 3cm outside the mat, where no amplification was observed, even when nested RT-PCR and multiple primer sets were employed (Fig. 4b; Fig. S7) [61], [62]. In the surficial sediment outside the mat, the lack of *mcrA* transcripts agrees with the absence of 16S rRNA transcripts from methane-cycling ANME archaea. Beneath the mat, transcripts of *mcrA* describe a similar population to that seen with 16S rRNA transcripts, containing multiple ANME-2 archaeal groups as well as group e, which has been found in similar methane seeps [44], [63], [64]. Although ANME-1 sequences were present in the 16S rRNA libraries, they were absent from the *mcrA* libraries, most likely because the primers used for *mcrA* are biased against ANME-1 (Table S2).

### Diversity Analysis

**Figure 5:**
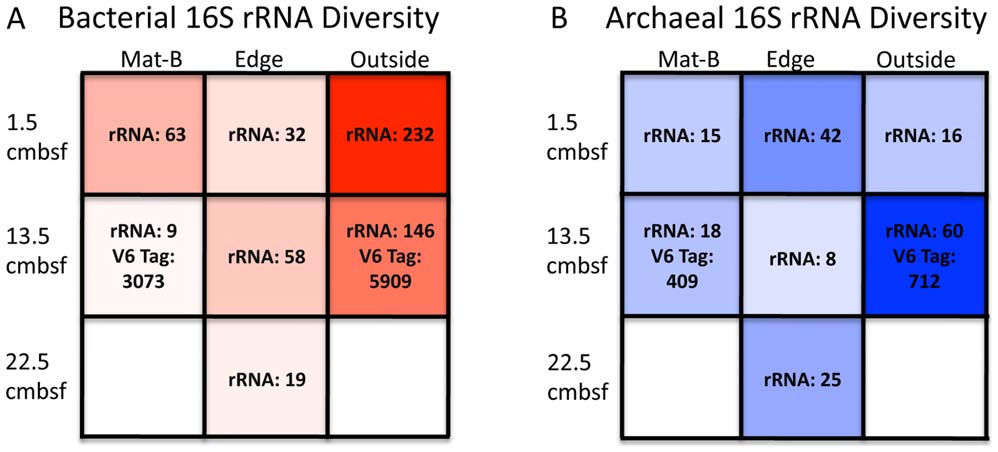
Diversity estimates of bacteria and archaea. Chao1 diversity estimates for rRNA clone libraries (rRNA) and where available for tag sequencing (V6 Tag) are listed for (A) bacteria and (B) archaea at different depths in the mat, at its edge, and just outside the mat. Transparency of each block corresponds to diversity relative to the highest diversity sample. Clone library sample sizes are the same as those in Figs. 3a and 4a.

Chao1 diversity estimates, based on 98% 16S rRNA similarity, ranged from 8 to 60 OTUs for archaea, and 9 to 232 OTUs for bacteria (Fig. 5). Chao1 diversity estimates based on V6-tag sequences at 97% OTU groupings, while higher, support the spatial trends predicted by the clone libraries. Sample sizes for clone libraries are in the range of 40 to 85 (Figs. 3a and 4a), whereas sample sizes for tags were 15,000 to 18,000 sequences.

In sediments under the mat, bacterial and archaeal Chao1 diversity estimates based on 16S rRNA were in similar ranges to each other (9–63 for bacteria, 8–42 for archaea). Outside the mat bacterial community richness was higher than that of archaea; it peaked in surface sediments outside the mat and decreased with depth outside the mat. All samples from under the mat had lower bacterial diversity than those from outside the mat. Archaeal community richness peaked at 12–15 cmbsf outside the mat. No consistent trends were seen for archaea relative to depth or presence of overlying mat.

## Discussion

### Correlation between Microbial Activity and Seeping Fluids

**Figure 6:**
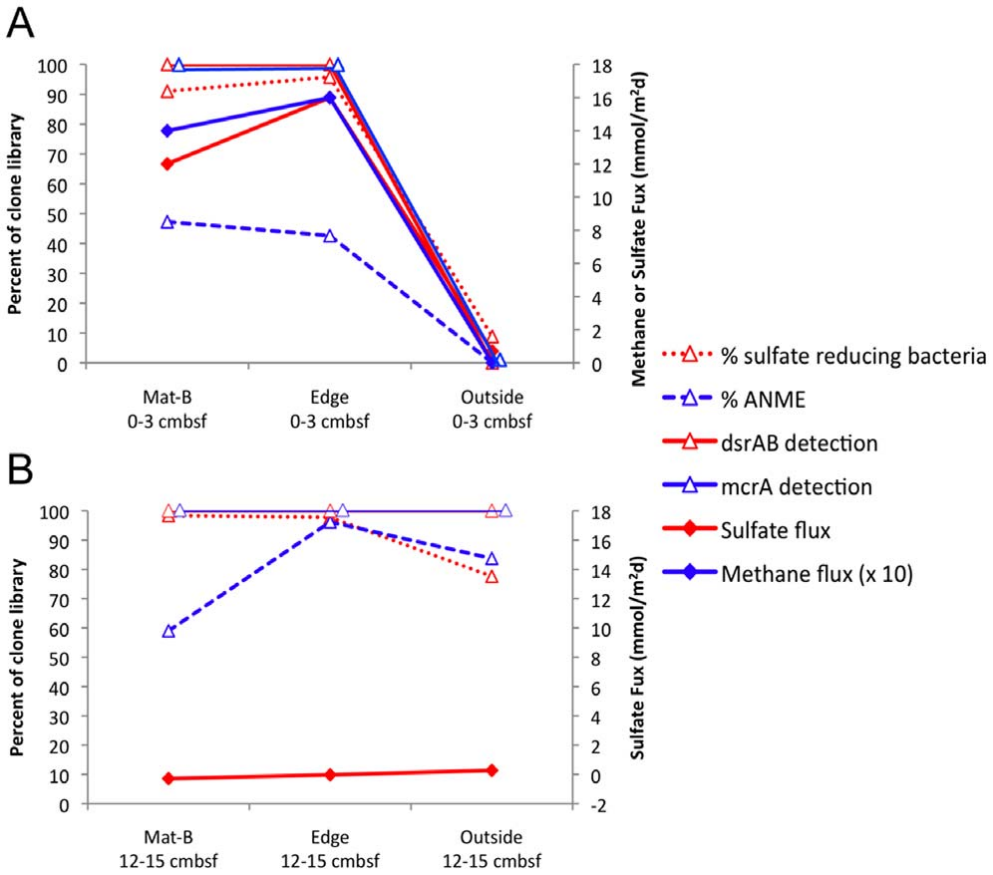
Correlation of geochemical and microbial stratification. Comparisons of geochemical fluxes and molecular microbiological data for three cores within a bacterial mat (Mat-B), at its edge (Edge), and less than a meter outside the mat (Outside). Plotted are percent of bacterial clones from putative sulfate reducing bacterial rRNA transcripts; percent of archaeal clones from putative methane oxidizer/methanogen ANME groups; presence or absence of *dsrAB* and *mcrA* mRNA clones (100%) or absence (0%), slightly offset from each other for visibility; the absolute value of the sulfate flux, since all were negative values; and methane flux multiplied by 10. Sulfate-related data is shown in red, and methane-related data is shown in blue. A) data for surface sediments (0–3 cmbsf), B) data for deeper sediments (12–15 cmbsf). Methane fluxes could not be accurately calculated for B) because methane concentrations were above atmospheric saturation and were not reliable.

Good correlation was observed in surface sediments between the composition of the active microbial community and geochemical processes. The abrupt decrease in sulfate flux and sulfate reduction rates just outside the mat was accompanied by a drop in the percentage of putative sulfate reducing groups in bacterial 16S rRNA-based clone libraries, as well as undetectable *dsrAB* transcripts (Fig. 6A). Correlation was also observed for methane flux, percentage of putative methane cyclers (ANME groups) in archaeal 16S rRNA transcript libraries, and *mcrA* in mRNA transcript clone libraries (Fig. 6A). Outside the mat, the bacterial community consists of phylotypes closely related to diverse aerobic, microaerophilic or nitrate-reducing bacteria, and the archaeal community is mostly composed of Marine Group I, whose cultured members are aerobic ammonia oxidizers and are commonly found in oxygenated seawater and sediments [65], [66]. Also, transcripts of *dsrAB* and *mcrA* were below detection limits with the primers used, which correlates well with the low sulfate flux and sulfate reduction rates. These trends show tight spatial coupling between subsurface processes, the active microbial community, and the presence of bacterial mat on the seafloor.

Deeper in the sediments underneath the mat, the tight spatial coupling between active microbial community and measured geochemical processes is absent (Fig. 6B). The transcript compositions of all three deep samples are similar to those of in-mat surficial sediments, but are not accompanied by high rates of sulfate reduction and methane oxidation (Figs. 2, 3, and 4). The phylotypes of sulfate reducers do not change after sulfate depletion at 12–15 cm under the mat and edge cores, suggesting that the dominant community composition does not change, even if their activity levels change in response to substrate limitation. Consistent retrieval of *dsrAB* genes in the deep sediments of all three cores is surprising since the sulfate reduction rates are so low that they do not cause a detectable decrease in sulfate concentrations with depth. Transcripts of *dsrAB* retrieved from deep samples would have had to persist in inactive cells for at least 161 years at 5 cm depth below the point where a sulfate flux is no longer measureable, assuming the maximum sedimentation rate for MC118 (31 cm/kyr [67]). Since such persistence of mRNA is extremely unlikely, we conclude that living sulfate-reducing populations are active at these depths. Sulfate reducers undergoing fermentation express *dsrAB* constitutively [34], so the presence of *dsrAB* may not necessarily indicate the occurrence of sulfate reduction. Alternately, sulfate could be recycled by reoxidation of sulfide coupled to iron reduction, described as “cryptic sulfate formation” in a study of Black Sea sediments [49]; however, this process would require the presence of a large bioavailable amount of a suitable electron acceptor such as iron to drive the sulfide reoxidation. Another possibility for deep sulfate regeneration is the occasional redistribution of nitrate (an electron donor for sulfide oxidation) to deep sediments through *Beggiatoa* spp., although we saw no direct evidence of the presence of *Beggiatoa* spp. deeper in the cores. Since ANME archaeal *mcrA* mRNA transcripts occur deeper in sediment cores where methane oxidation rates are very low, a small ANME population might survive on these low methane oxidation rates. Alternatively, since the edge core is net methanogenic (according to the δ^13^C profile of methane), ANME populations could switch to methanogenesis, as has been suggested previously [7].

Unlike surficial sediments, no large-scale changes were observed in the active microbial communities at 12–15 cmbsf between mat, edge, and outside cores (Fig. 6B). This deep sulfur cycling could therefore be the result of recent shifts in the location of vent conduits and the microbial population has yet to equilibrate with their new chemical environment. Seafloor observatories are needed to determine over what timescales these vent fluid shifts occur, and assess their relative importance in gene expression levels of the microbial community.

### Trends in Bacterial and Archaeal Diversity

Among the many possible controls that affect bacterial and archaeal diversity, we consider the roles of electron acceptor and carbon substrate availability in determining relative bacterial and archaeal diversity. Bacterial diversity was highest at 1.5 cmbsf outside the mat, suggesting the combined effects of pelagically-derived organic matter and energetically advantageous electron acceptors such as oxygen and nitrate. By comparison, all bacterial samples under the *Beggiatoa* spp. mat show decreased diversity, possibly a consequence of strongly reducing conditions in sulfate-reducing and methane-cycling sediments. The archaeal diversity trends do not show this strong contrast between sediments underneath the mat and outside the mat. Archaeal diversity peaked at 13.5 cmbsf outside the mat, suggesting an additive effect of overlapping surface and seep archaeal communities; 16S rRNA clone libraries show that pelagic Marine Group I archaea and anaerobic ANME archaea were both present. Under the mat, the active archaeal and bacterial communities showed similar diversity (Chao1 indices of 9 to 63 for bacteria vs. 8 to 42 for archaea), in contrast to the oxic sediments outside the mat that strongly favored bacterial diversity. In fact, three out of the five clone libraries under the mat and at its edge had higher archaeal than bacterial diversity. To our knowledge, this is the first documentation of higher archaeal than bacterial diversity in marine sediments. Bacterial diversity has generally been found to be much higher than archaeal diversity in a given environment [48], [68], although the discrepancy is much smaller in petroleum and natural gas seeps [68]. More research at a greater sampling depth is necessary to substantiate the higher archaeal than bacterial diversity, but these observations are consistent with the working hypothesis that archaea are low-energy specialists, and are widely adapted to highly reduced environments [69]. Other environmental studies substantiate this trend, with higher bacterial than archaeal diversity in microoxic mats [15] and seawater-mixed vent fluids [48].

### RNA Transcripts as Indicators of the Active Microbial Community

Our molecular analysis of bacterial and archaeal community structure and stratification has focused on the level of gene expression via RNA, not gene presence via DNA. Environmental microbial communities are most often studied by extracting, amplifying, and sequencing bulk DNA [70]. However, DNA-based clone libraries may not necessarily represent living microbes since extracellular DNA is preserved in cold anoxic environments [17], and eludes hydrolysis through adherence to mineral surfaces [71] from which it can nevertheless be amplified with PCR [72]. RNA, however, is an inherently less stable molecule than DNA, since it is mostly single-stranded and is susceptible to peptide backbone hydrolysis due to its extra 2′ hydroxyl group which stabilizes the transition state. For this reason, it has been used as an indicator of the potentially active microbial population [27], [44], [73], [74]. The short intracellular lifetime of messenger RNA (mRNA), and its direct link to metabolic processes makes it an even more promising indicator of microbial activity in environmental samples [33].

Indeed, we found that mRNA was more sensitive to environmental conditions than rRNA. In surface sediments outside the mat a few 16S rRNA transcripts from SRB were present, but these were not accompanied by *dsrAB* mRNA, as detectable by our primers. Unless these particular SRB have special adaptations for post-translational control of DSR protein, it is likely that they were not actively reducing sulfate. Given that the intracellular lifetime of *mcrA* mRNA molecules is on the scale of minutes and that of *dsrAB* is on the scale of days in laboratory cultures [31], [33], [34], and extracellular degradation is highly favorable, these molecules are likely indicators of active communities. Extraction of RNA directly from marine sediments is difficult given the often low activity (and therefore low mRNA copy number) of microbes in anoxic environments and the susceptibility of RNA to RNases during the extraction process [26]. Therefore, microbes that are in low abundance or those with low cellular RNA content were likely missed by our analysis. The differences in the compositions of 16S rRNA RT-PCR clone libraries and V6-tag sequences may be due to far greater sampling depth in the V6-tags, or differences in primer bias, so it is difficult to use these comparisons to infer differences in RNA- vs. DNA-based analyses. However, RNA-based studies allow access to the likely active members of the population and removes much of the uncertainty about the extent to which culture-independent methods describe functional populations.

### Relationship between Diversity Changes and Detection Limits

Apparent changes in diversity might be impacted by detection issues. For example, rare groups that are still detected in sediments with high biomass may fall below detection level in sediments that have a lower biomass, resulting in erroneously low diversity indices [75]. However, some of our archaeal results show the opposite trend, arguing that archaeal diversity trends are not an artifact of total sample size bias. The amount of archaeal 16S rRNA, estimated by dilution PCR [76] was highest in the surface outside the mat, a sample with low archaeal diversity.

Even if we assume equal detection sensitivity for different bacterial and archaeal groups, clone library representation can be read only as a relative, not as an absolute measure of their abundance. For example, what appears to be an increased contribution of sulfate reducing bacteria and ANME archaea at 12–15 cmbsf compared to the surface layer outside the mat, may instead reflect decreased contributions of surface-layer bacteria and archaea at depth, leaving SRB and ANMEs to comprise a larger percentage of the 12–15 cmbsf community. Finally, the 16S rRNA primers for bacteria and archaea are in principle subject to primer bias and mismatch problems [57]; however, 16S rRNA the primers used in this study were checked against phylum-level alignments of complete 16S rRNA genes, and they each detected a large number of lineages, including novel phylum-level bacterial lineages [47].

### Spatial Scales of Mat-Associated Biogeochemical Activity

One of the most interesting implications of this mat study is the extremely uneven spatial distribution of mat-associated microbial processes in surficial sediments at seep sites. Microbial mats cover only a small fraction of the total sediment surface area at methane and hydrocarbon seep sites. At MC118, *Beggiatoa* spp. mats are occasionally observed and recorded on JSL 2006 dive tapes, but an extensive video survey suggests that they cover only a small proportion of the seafloor, mostly in the northwestern crater of MC118, and to a lesser extent in the southeastern area [77]. At the same time they are hot spots of near-surface microbial sulfate-reducing and methane-oxidizing activity.

The mat in our current study appears to be anomalously large (∼10 m diameter) for this site, as no others of this size have been documented. A photomosaic survey of a limited area near the sampling site (Fig. 1c) indicates that only ∼1% of the sediment surface is covered with microbial mats. Randomly taken gravity cores from the wider MC118 area have yielded only a few cores (4 out of 30) with steep sulfate and methane gradients, although they were not covered by bacterial mat and had much deeper sulfate depletion depths (50–100 cmbsf) [3]. In addition to the rarity of mat-covered, active sediments, the measured rates for sulfate reduction and methane oxidation in surficial sediments within and outside of mats diverge by an order of magnitude. Depth-integrated sulfate reduction rates (± standard deviation) underneath the mat are 12.3±6.2 mmol m^−2^ d^−1^; they drop to 2.1±0.8 outside the mat (Fig. 2, Table S1). These values generally agree with those averaged from 3 different Gulf of Mexico white *Beggiatoa* spp. mats with no tubeworms (26.9±25.9 mmol m^−2^ d^−1^) [5]. Although mat-covered sediment accounts for only ∼1% of sediment area at MC118, depth-integrated sulfate reduction rates in mat-associated surficial sediments (upper 20–25 cm) are an order of magnitude higher than in surrounding sediments. The abrupt changes in methane concentrations by two orders of magnitude indicate similar variability in methane oxidation rates (in mat, 2.5±1.2 mmol m^−2^ d^−1^) and methanogenesis rates as well (Fig. 2). Thus, mat-covered sediments at MC118 have a disproportionately large contribution to microbial processes relative to their small areal coverage.

### Conclusions

High radiotracer rates of sulfate reduction, and methane oxidation, as well as steep methane and sulfate gradients in the center and edge of the *Beggiatoa* spp. mat suggest that the boundaries of rising methane-and hydrocarbon-rich fluids are delineated by overlying mat cover. Rates at the center and edge of the mat are nearly identical, and then drop sharply less than a meter outside the mat. These clear geochemical boundaries are reflected in the compositions of the active surface microbial community, with consistent community compositions of active sulfate reducers and methane-cycling microorganisms in the center and edge of the mat, but a large drop in their RNA expression levels immediately outside the mat.

The deeper microbial communities outside the mat, however, look more similar to those under the mat. Therefore visually undistinguished sediments without conspicuous mat cover (and no porewater evidence for hydrocarbon seepage) can still harbor anaerobic methane- and sulfur-cycling communities that express genes for metabolic activities, but remain below detection limit in the geochemical measurements. High levels of sulfate reduction and methane oxidation in these sediments could resume quickly at the onset or reintroduction of active seeping, resulting in sulfide production and the rapid development of *Beggiatoa* spp. mats. As a result, microbial mat formation and the establishment of a sulfur-and methane-cycling, mat-associated microbial community in surficial sediments would be rapid and accessible to continuous *in-situ* observation over days and weeks [14].

These results validate that the often-observed patchiness and small-scale spatial architecture of microbial mats and methane seeps correspond to a profound reorganization of microbial community composition, activity patterns and geochemical imprint on spatial scales of tens of centimeters both vertically and horizontally in the sediments. Microbial mats play an important role as indicators of subsurface microbial heterogeneity and activity, a role proposed previously for seafloor fauna [78]. Systematic recording and documentation of visible seafloor heterogeneity and microbial mats over small spatial scales is therefore an essential component of microbial habitat studies and of foremost importance for sampling designs that capture the fundamental characteristic of microbial habitat patchiness.

## Methods

### Site Description and Sampling

Mississippi Canyon Block 118 (MC118) in the Gulf of Mexico is characterized by seafloor-breaching methane hydrate deposits and thermogenic hydrocarbon-rich fluids pushed upwards through fractures in the sediments by salt domes [79]. It is located offshore of Louisiana in ∼890 m of water at 5.5°C bottom water temperature (28°51.47, 88°29.52) (Fig. 1b). In September 2006 using the Johnson-Sea-Link submersible, four push-cores were taken across a wide (∼10 m) white seafloor microbial mat: two near the center of the mat less than a meter away from each other, one at the edge of the mat (∼10 cm from uncovered sediment, and ∼50 cm outside the mat. The two cores taken from the center of the mat were underlain by a hard surface. Gas bubbles were fizzing from cores taken from the mat and edge of the mat upon arrival at the ship, but the core from outside the mat was undisturbed. In a shipboard 4°C room, the cores were sub-sectioned into 3 cm intervals, and microbiological samples were taken in sterile 30 ml cut-off syringes and frozen immediately in liquid nitrogen. From each interval, subsamples were taken for porewater geochemistry and radiotracer rates. Only the mat and margin mat cores, not the outside mat core, smelled sulfidic.

The mosaic was generated from a self-contained digital still stereo camera package developed at the Australian Centre for Field Robotics. The camera system was mounted on the Johnson-Sea-Link II and used to acquire 12 bit, 1.4 Mpixel imagery at 1 Hz from an altitude of 3 to 4 m from the seafloor. The speed of the submersible was such that high overlap (over 75%) was typical. The imagery was assembled into a composite view using the approach described in [80].

### Porewater Geochemical Analysis

For sulfate measurements, plastic 15 ml tubes filled completely with sediment were centrifuged and the resulting porewater was filtered at 0.2 µm, acidified with 10% HCl, and measured shipboard using a 2010i Dionex ion chromatograph (Sunnyvale, CA), as previously described [81]. For methane measurements, 4 ml sediments were added to 60 ml serum vials containing 1 ml 0.1 M KOH, and were stoppered and crimp-sealed. A 5 mL headspace aliquot was analyzed on a Shimadzu Mini II gas chromatograph (Kyoto, Japan) equipped with flame ionization detector. Carbon stable isotope ratios for dissolved methane were obtained using a pre-concentrating system on-line with a continuous flow 5890 Hewlett-Packard gas chromatograph (Palo Alto, CA), capillary combustion, and isotope ratio mass spectrometry as described in Rice et al. [82]. Results are reported using the standard “del” notation, δ^13^C (‰) = [R_(sample)_/R_(PDB standard)_–1]*1000, where R is the ratio of the heavy to light isotope relative to the Pee Dee Belemnite standard [83]. The precision for replicate measurements of single samples was ±3 percent for sulfate, chloride, and methane concentrations. Sulfate reduction rate and methane oxidation rate measurements were made as previously described [84]. These rate methods measure only methane oxidation and not methanogenesis; the direction of the net reaction can only be gleaned from trends of δ^13^C of methane with depth.

A 1-D, inverse, reaction-transport model was used to compare concentration profiles to radiotracer rate measurements based on the following equation [41], [85]:
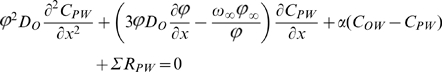
where ϕ is porosity, D_O_ is molecular diffusivity, C_PW_ is the concentration of the solute in sediment porewater, x is the depth interval in the sediment, ω is the sedimentation rate, α is the bioirrigation coefficient, C_PW_ is the concentration of the solute in the overlying water, and R_PW_ is the reaction rate of the porewater constituent. The first term in the equation accounts for molecular diffusion, the second for sedimentation and compaction, the third for bioirrigation, and the fourth for reaction rate.

### RNA Extraction, Amplification, Cloning and Sequencing

Total RNA was extracted following previously described methods [74], [86], from the following samples: Mat-B (0–3 cmbsf), Mat-B (12–15 cmbsf), Edge (0–3 cmbsf,) Edge (12–15 cmbsf,) Edge (21–24 cmbsf), Outside (0–3 cmbsf), and Outside (12–15 cmbsf). Briefly, ∼4 ml sediment was mixed with 5 ml phenol (pH 5), 5 ml of extraction buffer (50 mM sodium acetate and 10 mM EDTA, pH 5), and 0.5 ml 20% SDS. This mixture was bead-beaten with 0.1 mm silica beads, then extracted sequentially with phenol, phenol-chloroform (1∶1), and chloroform, precipitated in 7.5 mM ammonium acetate mixed with isopropanol, and washed with 80% ethanol. The pellet was resuspended in water and incubated with 4 µl TurboDNase at 37°C for 30 minutes, followed by purification with the Qiagen RNeasy MinElute kit.

Bacterial 16S rRNA cDNAs were amplified with B8f-B1492r [47] with an annealing temperature of 60°C for Mat-B (0–3 cmbsf), Mat-B (12–15 cmbsf), and Edge (0–3 cmbsf) and 58°C for the rest; *dsrAB* transcript cDNAs were amplified with DSR1f-DSR4r [87] at an annealing temperature of 54°C with a nested reamplification with 1f1r [50] at 48°C; and *mcrA* transcript cDNA were amplified with ME1-ME2 [61] at an annealing temperature of 55°C. For amplification of archaeal 16S rRNA genes, A8f and A1492r [47] were used at an annealing temperature of 59–60°C. In the samples from outside the mat, initial amplification using these primers had to be followed by nested reamplification with primer combination A21f-A915r [88], [89] at an annealing temperature of 58°C in order to see a product on a 1.5% agarose gel. Primer sequences and known mismatches are listed in Table S2. All reverse transcription and PCR reactions took place in a single tube using the reverse primer as the reverse transcription primer.

Each 25 µl RT-PCR reaction contained 1 µl RNA template, 0.15 µl each primer solution (100 pmol/µl), 1 µl bovine serum albumin (10 mg/ml; absent in bacteria reactions), as well as the following products from the Takara OneStep RT-PCR kit Version 3.0: 12.5 µl buffer, 0.5 µl RNase inhibitor, 0.5 µl HotStar Taq, and 0.5 µl reverse transcriptase. Each 25 µl nested PCR reaction contained 1 µl cDNA template, 0.15 µl each primer solution (100 pmol/µl), 1 µl bovine serum albumin (10 mg/ml), 4 µl deoxynucleotide triphosphate (10 mM each dATP, dCTP, dGTP and dTTP), 2.5 µl 10× FastBuffer I (Takara), and 0.125 µl SpeedStar Taq (Takara).

Conditions for RT-PCR in a Bio-Rad iCycler (Hercules, CA) were as follows: reverse transcription at 42°C for 15 min, reverse transcriptase inactivation and polymerase activation at 95°C for 2 min, followed by 25 cycles for bacterial 16S rRNA cDNA and archaeal 16S rRNA cDNA and 40 cycles for *dsrAB* mRNA cDNA and *mcrA* mRNA cDNA, each consisting of 5 s denaturation at 95°C, 15 s at primer annealing temperature (see above), and 20 s elongation at 72°C, plus a final elongation at 72°C for 10 min. Nested PCR for *dsrAB* required the following protocol: 94°C polymerase activation for 2 min, followed by 40 cycles of 98°C denaturation for 10 s, 48°C annealing for 15 s, and 72°C extension for 20 s, plus a final elongation at 72°C for 10 min. All PCR and RT-PCR products were purified using either a MoBio PCR Clean-up kit or purification in a 1% agarose gel and MoBio UltraSpin for gel purification. Purified products were cloned using the TOPO TA PCR cloning Kit, and transformed into *E. coli* by electroporation following the manufacturer's protocols (Invitrogen, San Diego, California). Sequences were obtained at the Josephine Bay Paul Center at the Marine Biological Laboratory (Woods Hole, MA), using an ABI 3730 sequencer, or at Genewiz (South Plainfield, NJ) on an ABI Prism 3730xl sequencer. Vector and primer sequences were removed from sequences and forward and reverse reads were assembled into contigs using Sequencher 4.7. Ribosomal sequences were aligned against the 2007 Silva release with ARB (www.arb-home.de). Sequences were deposited in NCBI Genbank with accession numbers GU190968-GU191015 for archaeal 16S, GU302419-GU302497 for bacterial 16S, GU302498-GU302509 for *mcrA*, and GU302510-GU302521 for *dsrAB*.

### Tag Sequencing

DNA was extracted from 0.5 g of sediment using the MoBio DNA Power Soil Kit (MoBio Inc, Carlsbad, CA). Using the methods of the International Census of Marine Microbes (ICoMM), the variable 6 (V6) region of the 16S rRNA gene was amplified and subjected to 454 pyrosequencing on a Roche GS20. All PCR methods, primers and analysis tools are detailed on the ICoMM website (www.vamps.mbl.edu; see also 17). Quality control included removing sequences with ambiguous base calls, or ones that did not match the primers perfectly [90]. Chao1 estimates are shown at 3% OTU clustering, therefore insertions and deletions of individual bases during amplification or pyrosequencing did not contribute to diversity estimates. Tag sequences are publicly available from http://vamps.mbl.edu as the following datasets: GMS_0003_2006_09_14 (bacteria, under mat), GMS_0004_2006_09_14 (archaea, under mat), GMS_0005_2006_09_14 (bacteria, outside mat) and GMS_0006_2006_09_14(archaea, outside mat).

### Controls on RT-PCR

Reverse transcriptase-free control RT-PCR reactions were made for each clone library to check for the co-extraction of DNA. No PCR products were visible on a 1.5% agarose gel for any of the controls. In order to check for PCR products not visible in the gel, nine No RT controls from six different RNA extractions were gel purified, cloned, and sequenced. Less than 10% of plasmids contained any inserts, and of those that did, most were plasmid DNA or other bits of DNA not present in any of the RT-PCR clone libraries. Three clone libraries contained 4 clones total of Eel-2 Bacterial 16S rRNA gene sequences identical to the most numerous clone in RT-PCR clone libraries. However, since 1) these PCR products were gel purified alongside concentrated RT-PCR products used to guide the cutting of the invisible bands, and 2) two of the three No RT clone libraries were made with *dsrAB* primers, not bacterial 16S rRNA ones, it is likely that this small number of sequences were contamination from RT-PCR products during gel-cutting. Extraction blanks were also carried through all stages of RNA extraction, purification, RT-PCR, and nested PCR, where appropriate. No extraction blanks were visible on gels for any clone library.

### Sequence Analysis

Operational taxonomic units (OTUs) were determined by aligning 500–600bp of each forward read in ClustalX, and grouping into 99% similar OTUs using a distance matrix generated in PAUP4.b10 [91]. Representatives of each OTU were reverse sequenced to get a full-length read. Chimeras were identified using Pintail and also by Blasting 5′ and 3′ ends separately to check for agreement. Full-length and short reads were then aligned using ARB (www.arb-home.de), and phylogenetic groups were determined. Only full-length reads were included in the phylogenetic trees, which were made in PAUP. Chao1 diversity estimates were calculated using the methods of DOTUR [92], which are based on the EstimateS modification [93] of the original Chao1 diverisity estimator [94]. Chao1 values (S_Chao1_) were calculated with a bias correction for the presence of singletons as S_Chao1_ = S_obs_+n_1_ (n_1_−1)/(2*(n_2_+1)), where S_obs_ is the observed number of species, n_1_ is the number of OTUs with only one sequence, and n_2_ is the nmber of OTUs with only two sequences. Chao1 is a method for predicting actual diversity, assuming that only a subset of the total population has been sampled; and works well at a low average sample capture probability [95]. All samples except for archaea Edge 21–24 cmbsf and bacteria Edge 0–3 cmsf and Edge 12–15 cmbsf deviated from the average clone library size by less than 20% of their total value (clone library sample sizes are listed in Figs. 3a and 4a).

## Supporting Information

Table S1 Comparison of depth-integrated sulfate reduction and methane oxidation rates (mmol m-2 d-1) to concentrations fluxes of sulfate and methane (mmol m-2 d-1), respectively.
(0.04 MB DOC)

Table S2 Primer sequences used in the study, their annealing temperatures, target groups, and known mismatches.
(0.07 MB DOC)

Figure S1 Model fit to sulfate concentration data (red line), or sulfate reduction rate data (blue line) for Edge core. Yellow markers are the data from Figure 2c.
(9.28 MB TIF)

Figure S2 Neighbor-joining tree of cDNA of full-length Deltaproteobacterial 16S rRNA sequences for all samples. The nodes are labeled with parsimony-based boostrap values (1000 repetitions) that were over 60%. OTUs are based on 98% similarity. Sequences from dive 3570 are in colors corresponding to those of Fig. 3a groupings. Clones given their core name (either MatB, Edge, or Out) followed by the beginning of their depth interval (0–3 cmbsf, 12–15 cmbsf, or 21–24 cmbsf), the type of cDNA (arc or bac for archaeal or bacterial 16S rRNA, mcr or dsr for mRNA), and a unique clone number. The number of sequences included in each OTU are in parentheses after the clone name, with the core and depth listed.
(10.24 MB TIF)

Figure S3 Neighbor-joining tree of cDNA of full-length non-Deltaproteobacterial 16S rRNA sequences for all samples. The nodes are labeled with parsimony-based boostrap values (1000 repetitions) that were over 60%. OTUs are based on 98% similarity. Sequences from dive 3570 are in colors corresponding to those of Fig. 3a groupings.
(0.66 MB TIF)

Figure S4 Comparison of Blast hits for sequence tags from V6 tag pyrosequencing and 16S rRNA sequences from RT-PCR clone libraries for 2 samples (12–15 cmbsf in Mat-B, and 12–15 cmbsf Out). Shown are 100% bar charts for A) bacteria and B) archaea.
(9.72 MB TIF)

Figure S5 Neighbor-joining tree of amino acid translations of dsrAB transcripts for all samples. The nodes are labeled with parsimony-based boostrap values (1000 repetitions) that were over 60%. Sequences from dive 3570 are in colors corresponding to those of Fig. 3b groupings.
(5.47 MB TIF)

Figure S6 Neighbor-joining tree of cDNA of full-length archaeal 16S rRNA sequences for all samples. The nodes are labeled with parsimony-based boostrap values (1000 repetitions) that were over 60%. OTUs are based on 98% similarity. Sequences from dive 3570 are in colors corresponding to those of Fig. 4a groupings.
(5.38 MB TIF)

Figure S7 Neighbor-joining tree of amino acid translations of mcrA transcripts for all samples. The nodes are labeled with parsimony-based boostrap values (1000 repetitions) that were over 60%. Sequences from dive 3570 are in colors corresponding to those of Fig. 4b groupings.
(7.09 MB TIF)
