# Supplementary material for: Spatial Structure and Activity of Sedimentary Microbial Communities Underlying a Beggiatoa spp. Mat in a Gulf of Mexico Hydrocarbon Seep
Source: PLoS One. 2010 Jan 15;5(1):e8738. doi: 10.1371/journal.pone.0008738 (PMC2806916; doi:10.1371/journal.pone.0008738)
Supplement: Table S1 — Comparison of depth-integrated sulfate reduction and methane oxidation rates (mmol m-2 d-1) to concentrations fluxes of sulfate and methane (mmol m-2 d-1), respectively. (0.04 MB DOC) [file pone.0008738.s001.doc]

|  | **Mat-A** | **Mat-A** | **Mat-B** | **Mat-B** | **Edge** | **Edge** | **Outside** | **Outside** |
| --- | --- | --- | --- | --- | --- | --- | --- | --- |
| **Measurement** | **1^st^** | **2^nd^** | **1^st^** | **2^nd^** | **1^st^** | **2^nd^** | **1^st^** | **2^nd^** |
| **Sulfate Reduction** | |  |  |  |  |  |  |  |
| Integrated rate | 9.3 | 11.1 | 11.1 | 4.0 | 22.3 | 15.9 | 1.5 | 2.7 |
| Flux | 17.9 |  | 12.0 |  | 16.0 |  | 0.7 |  |
| **Methane Oxidation** | |  |  |  |  |  |  |  |
| Integrated rate | 3.2 | 4.2 | 1.1 | 1.3 | 2.9 | 2.1 |  |  |
| Flux | 6.4 |  | 1.4 |  | 1.6 |  |  |  |

^a^ Flux calculations were made using Fick’s law, with modifications [1]: J = φ^3^D_s_(dC/dx)

Where J is the total flux, φ is the porosity (0.9); D_s_ (5.883 x 10^-6^ cm^2^/s for sulfate and 9.511 x 10^-6^ cm^2^/s for methane) is the diffusion coefficient for the solutes at in situ depth (900 m), temperature (5.5°C), and salinity (35 ppt); dC/dx is the change in sulfate or methane concentration divided by the depth interval.

1. Ullman WJ, Aller RC (1982) Diffusion coefficients in nearshore marine sediments. Limnology and Oceanography 27: 552-556.
