# Supplementary material for: Spatial Structure and Activity of Sedimentary Microbial Communities Underlying a Beggiatoa spp. Mat in a Gulf of Mexico Hydrocarbon Seep
Source: PLoS One. 2010 Jan 15;5(1):e8738. doi: 10.1371/journal.pone.0008738 (PMC2806916; doi:10.1371/journal.pone.0008738)
Supplement: Table S2 — Primer sequences used in the study, their annealing temperatures, target groups, and known mismatches. (0.07 MB DOC) [file pone.0008738.s002.doc]

| Primer | Target cDNA | Predicted target group | Known mismatches^a^ | Sequence (5’ to 3’) | Annealing temp (°C) | Ref |
| --- | --- | --- | --- | --- | --- | --- |
| A8f | 16S | Archaea | unknown | TCC GGT TGA TCC TGC C | 58 | [1] |
| A915r | 16S | Archaea | Uncultured archaea^b^ | GTG CTC CCC CGC CAA TTC CT | 58 | [2] |
| A1492r | 16S | Archaea | unknown | GGC TAC CTT GTT ACG ACT T | 58 | [1] |
| B8f | 16S | Bacteria | unknown | AGR GTT TGA TCC TGG CTC AG | 58 | [1] |
| B1492r | 16S | Bacteria | unknown | CGG CTA CCT TGT TAC GAC TT | 58 | [1] |
| ME1 | mcrA | ANME/ME | ANME-1^c^ | GCM ATG CAR ATH GGW ATG TC | 55 | [3] |
| ME2 | mcrA | ANME/ME | ANME-1^c^ | TCA TKG CRT AGT TDG GRT AGT | 55 | [3] |
| Dsr1f | dsrA | SRB | None reported^d^ | ACS CAY TGG AAG CAC G | 54 | [4,5] |
| Dsr4r | dsrB | SRB | None reported^d^ | GTG TAG CAG TTA CCG CA | 54 | [4,5] |
| 1f1 | dsrA | SRB | Many Desulfobulbaceae | CAG GAY GAR CTK CAC CG | 48 | [6] |
| 1r1 | dsrB | SRB | No major groups | CCC TGG GTR TGR AYR AT | 48 | [6] |

R = A/G, M = A/T, H = A/C/T, W = A/T, K = G/T, D = A/G/T, S = G/C, Y = C/T

^a^Mismatches to Genbank archived sequences. Some sequences have unknown mismatches since 3’ and 5’ ends of most environmental 16S sequences in Genmank remain unsequenced.

^b^Specific groups listed in [7].

^c^Fosmid sequences containing *mcrA* genes were obtained from Genbank from [8] and 5 out of 5 ANME-1 fosmids had 4 mismatches to ME1 and 2 mismatches to ME2 (GZfos11H11, GZfos13E1, GZfos24D9, GZfos18B6, GZfos17A3); 1 ANME-2 fosmid had either no mismatches to ME1 and 1 mismatch to ME2 (GZfos35D7); or 1 mismatch to ME1 and 2 mismatches to ME2 (GCfos26B2). A large *mcrA* database comparison is in [9].

^d^Dsr1f-Dsr4r amplified all phyla of cultured sulfate reducing bacteria.

1. Teske A, Hinrichs K-U, Edgcomb V, de Vera Gomez A, Kysela D, et al. (2002) Microbial diversity of hydrothermal sediments in the Guaymas Basin: evidence for anaerobic methanotrophic communities. Applied and Environmental Microbiology 68: 1994-2007.

2. DeLong EF (1992) Archaea in coastal marine environments. Proceedings of the National Academy of Sciences USA 89: 5685-5689.

3. Hales BA, Edwards C, Ritchie DA, Hall G, Pickup RW, et al. (1996) Isolation and identification of methanogen-specific DNA from blanket bog peat by PCR amplification. Applied and Environmental Microbiology 62: 668-675.

4. Wagner M, Roger AJ, Flax JL, Brusseau GA, Stahl DA (1998) Phylogeny of dissimilatory sulfite reductases supports an early origin of sulfate respiration. Journal of Bacteriology 180: 2975-2982.

5. Klein M, Friedrich M, Roger AJ, Hugenholtz P, Fishbain S, et al. (2001) Multiple lateral transfers of dissimilatory sulfite reductase genes between major lineages of sulfate-reducing prokaryotes. Journal of Bacteriology 183: 6028-6035.

6. Dhillon A, Teske A, Dillon J, Stahl DA, Sogin ML (2003) Molecular characterization of sulfate-reducing bacteria in the Guaymas Basin. Applied and Environmental Microbiology 69: 2765-2772.

7. Teske A, Sørensen KB (2008) Uncultured archaea in deep marine subsurface sediments: have we caught them all? The ISME Journal 2: 3-18.

8. Hallam SJ, Putnam N, Preston CM, Detter JC, Rokhsar D, et al. (2004) Reverse methanogenesis: testing the hypothesis with environmental genomics. Science 305: 1457-1462.

9. Lever MA (2008) Anaerobic carbon cycling pathways in the deep subseafloor investigated via functional genes, chemical gradients, stable carbon isotopes, and thermodynamic calculations. Chapel Hill, NC: University of North Carolina.
