# Supplementary figures and images for: Spatial Structure and Activity of Sedimentary Microbial Communities Underlying a Beggiatoa spp. Mat in a Gulf of Mexico Hydrocarbon Seep
Source: PLoS One. 2010 Jan 15;5(1):e8738. doi: 10.1371/journal.pone.0008738 (PMC2806916; doi:10.1371/journal.pone.0008738)

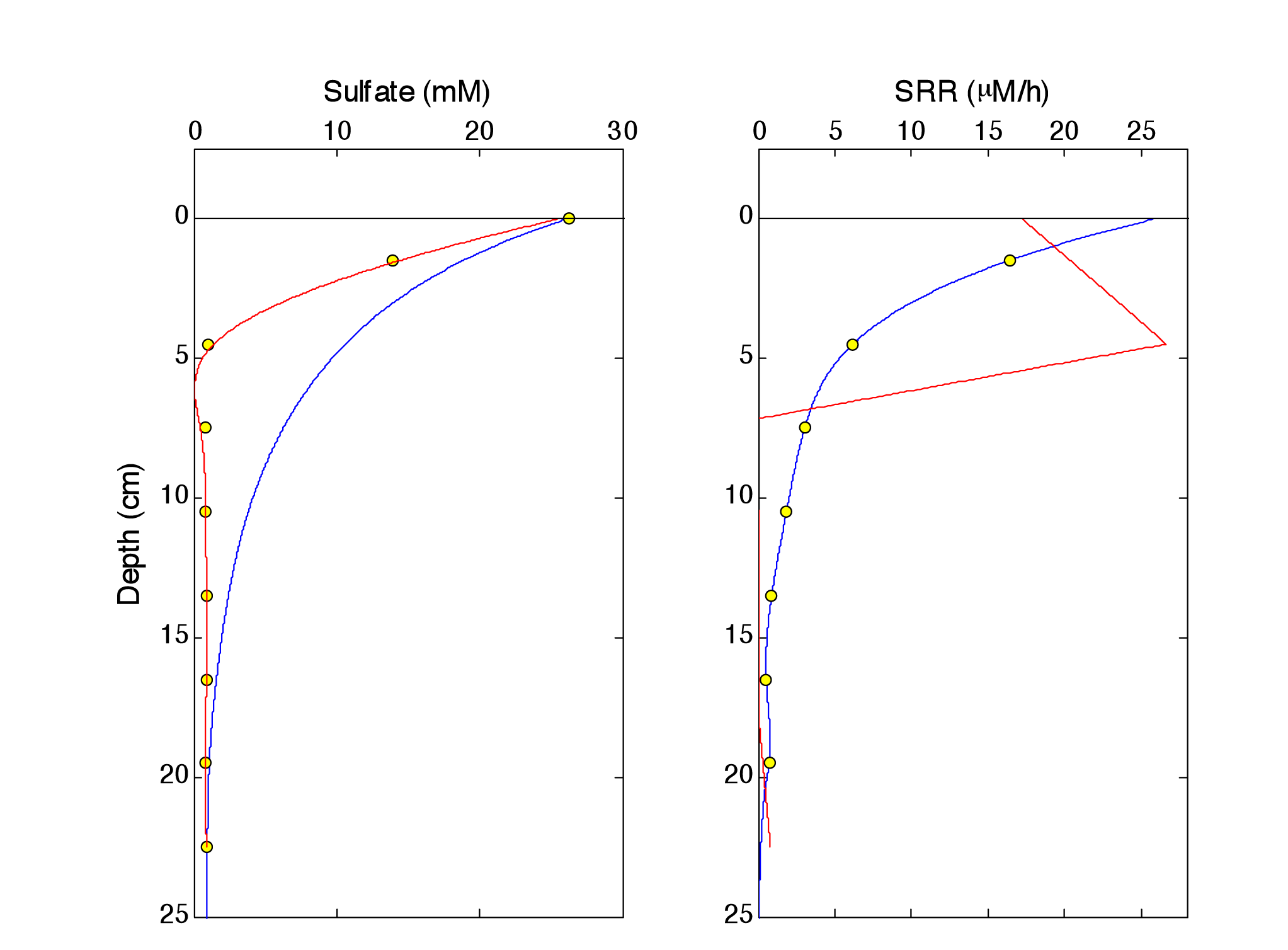

Supplement: Figure S1 — Model fit to sulfate concentration data (red line), or sulfate reduction rate data (blue line) for Edge core. Yellow markers are the data from Figure 2c. (9.28 MB TIF) [file pone.0008738.s003.tif]

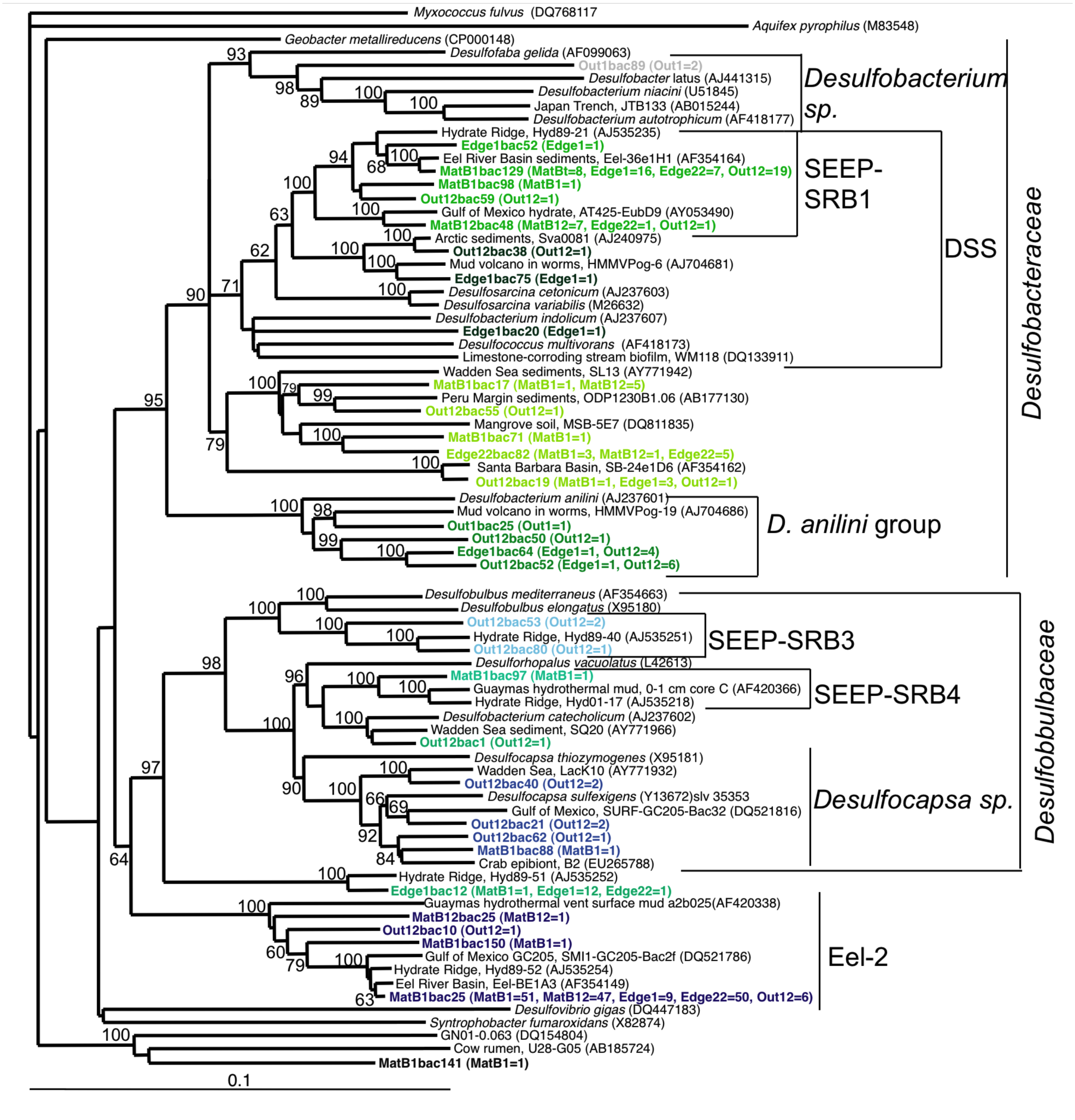

Supplement: Figure S2 — Neighbor-joining tree of cDNA of full-length Deltaproteobacterial 16S rRNA sequences for all samples. The nodes are labeled with parsimony-based boostrap values (1000 repetitions) that were over 60%. OTUs are based on 98% similarity. Sequences from dive 3570 are in colors corresponding to those of Fig. 3a groupings. Clones given their core name (either MatB, Edge, or Out) followed by the beginning of their depth interval (0–3 cmbsf, 12–15 cmbsf, or 21–24 cmbsf), the type of cDNA (arc or bac for archaeal or bacterial 16S rRNA, mcr or dsr for mRNA), and a unique clone number. The number of sequences included in each OTU are in parentheses after the clone name, with the core and depth listed. (10.24 MB TIF) [file pone.0008738.s004.tif]

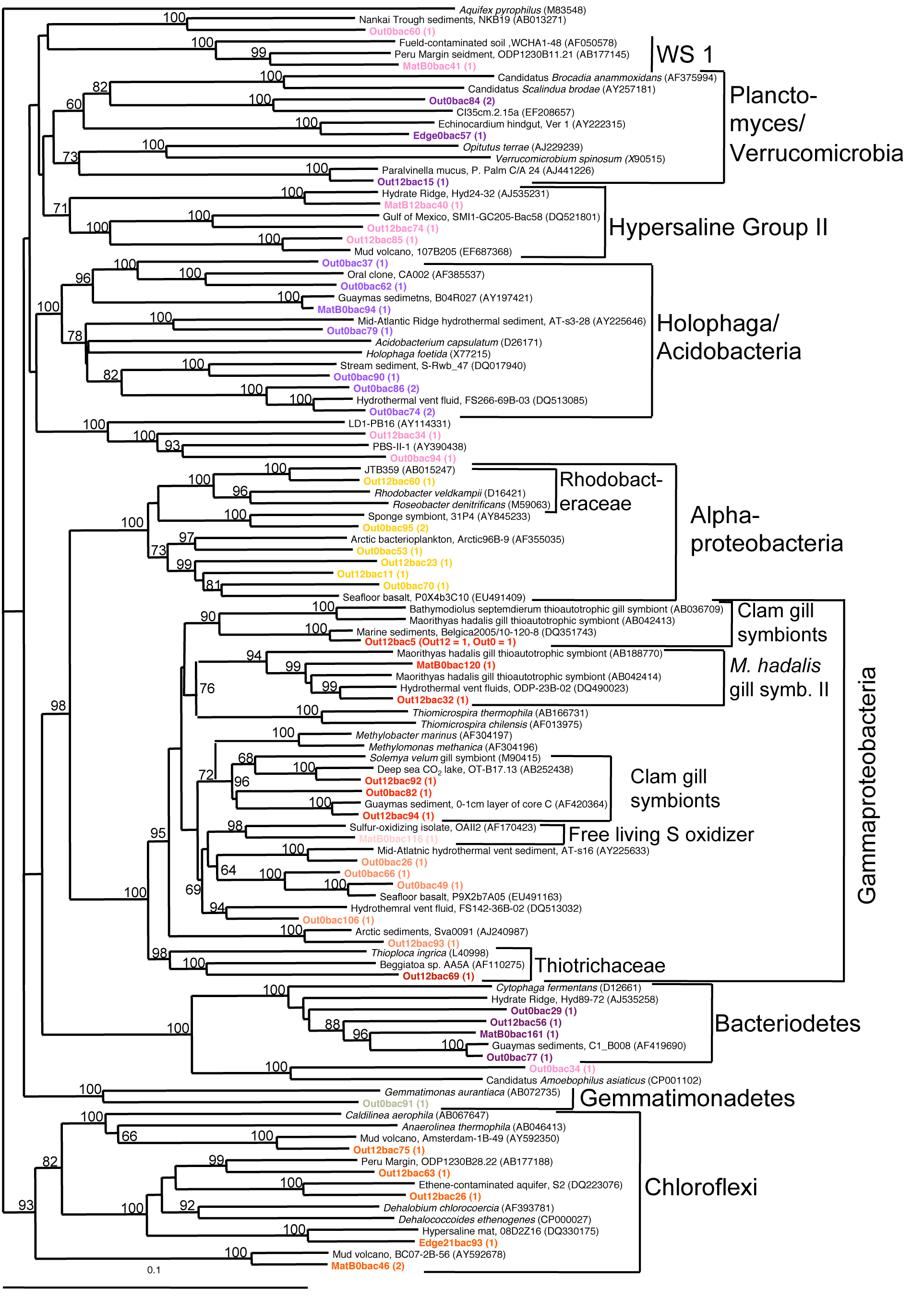

Supplement: Figure S3 — Neighbor-joining tree of cDNA of full-length non-Deltaproteobacterial 16S rRNA sequences for all samples. The nodes are labeled with parsimony-based boostrap values (1000 repetitions) that were over 60%. OTUs are based on 98% similarity. Sequences from dive 3570 are in colors corresponding to those of Fig. 3a groupings. (0.66 MB TIF) [file pone.0008738.s005.tif]

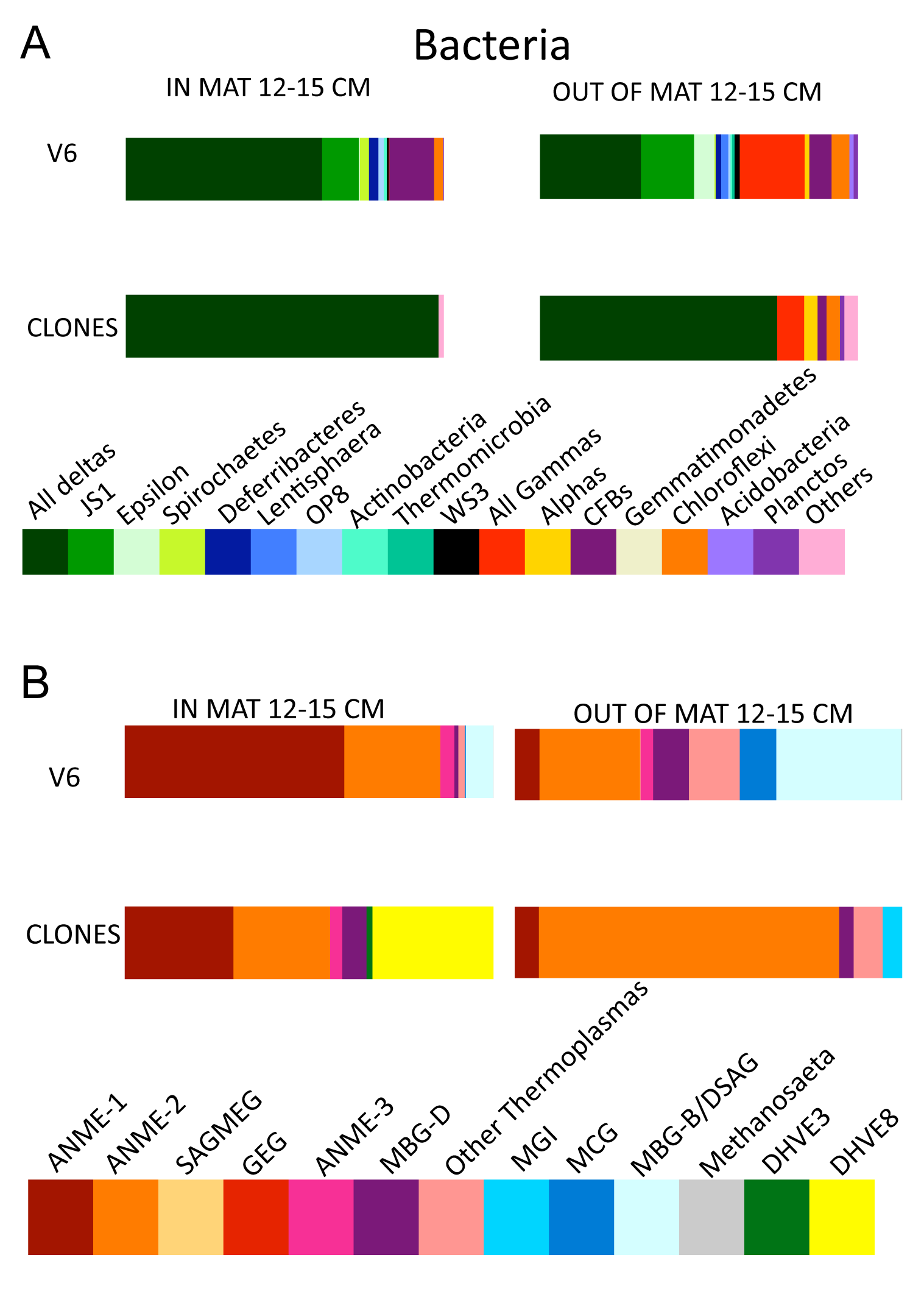

Supplement: Figure S4 — Comparison of Blast hits for sequence tags from V6 tag pyrosequencing and 16S rRNA sequences from RT-PCR clone libraries for 2 samples (12–15 cmbsf in Mat-B, and 12–15 cmbsf Out). Shown are 100% bar charts for A) bacteria and B) archaea. (9.72 MB TIF) [file pone.0008738.s006.tif]

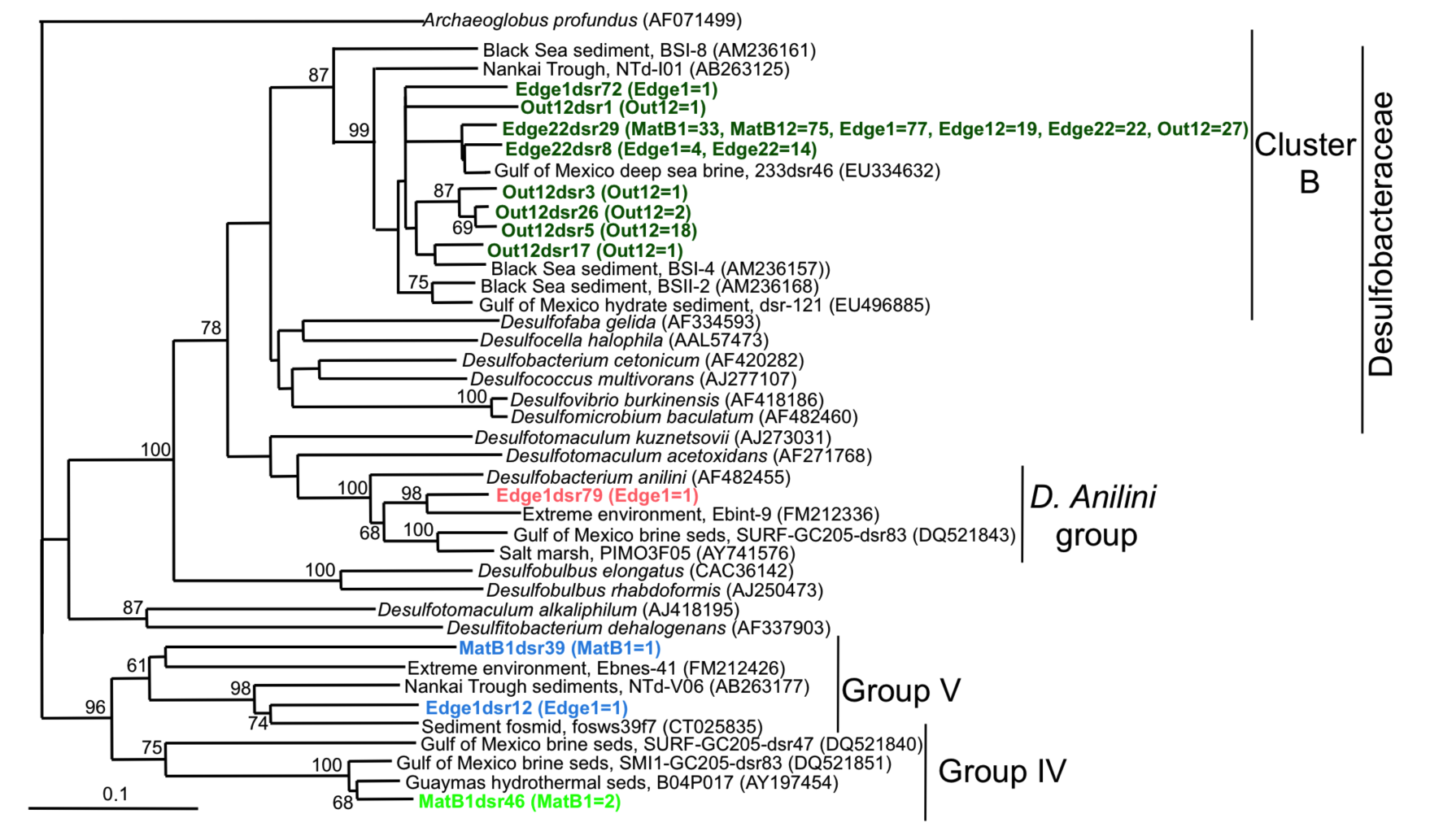

Supplement: Figure S5 — Neighbor-joining tree of amino acid translations of dsrAB transcripts for all samples. The nodes are labeled with parsimony-based boostrap values (1000 repetitions) that were over 60%. Sequences from dive 3570 are in colors corresponding to those of Fig. 3b groupings. (5.47 MB TIF) [file pone.0008738.s007.tif]

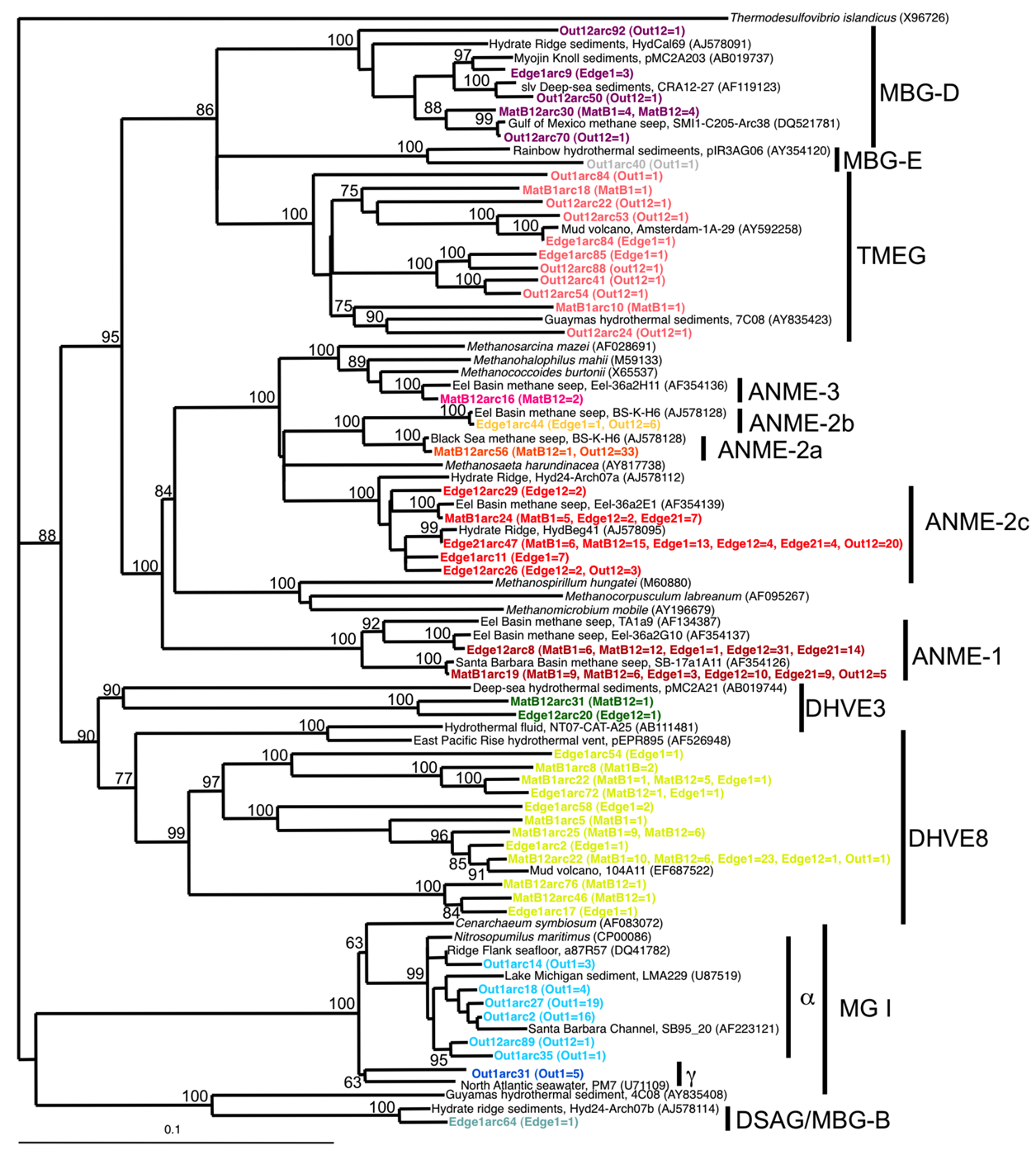

Supplement: Figure S6 — Neighbor-joining tree of cDNA of full-length archaeal 16S rRNA sequences for all samples. The nodes are labeled with parsimony-based boostrap values (1000 repetitions) that were over 60%. OTUs are based on 98% similarity. Sequences from dive 3570 are in colors corresponding to those of Fig. 4a groupings. (5.38 MB TIF) [file pone.0008738.s008.tif]

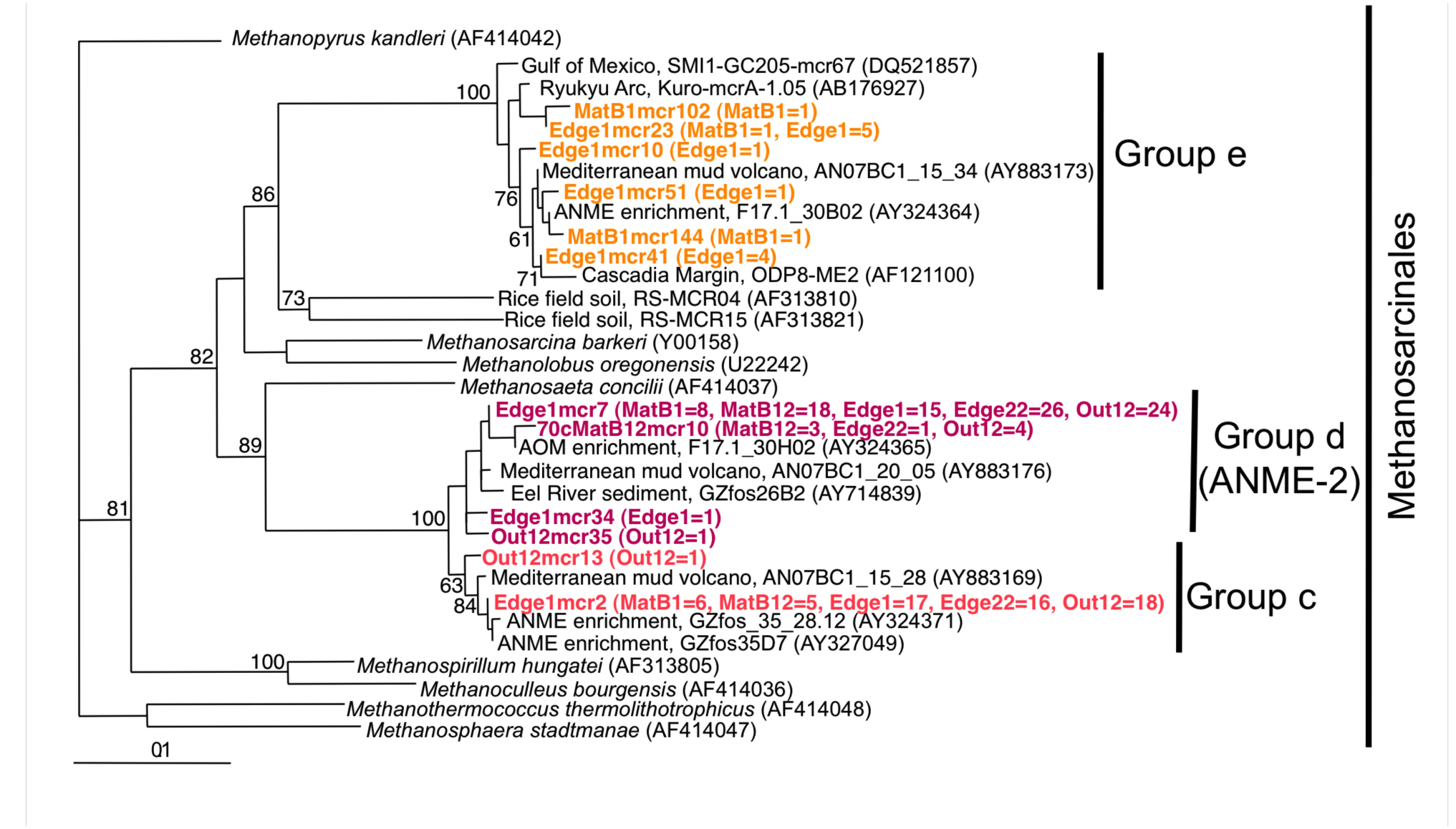

Supplement: Figure S7 — Neighbor-joining tree of amino acid translations of mcrA transcripts for all samples. The nodes are labeled with parsimony-based boostrap values (1000 repetitions) that were over 60%. Sequences from dive 3570 are in colors corresponding to those of Fig. 4b groupings. (7.09 MB TIF) [file pone.0008738.s009.tif]
